# Using a conversational agent for thought recording as a cognitive therapy task: Feasibility, content, and feedback

**DOI:** 10.3389/fdgth.2022.930874

**Published:** 2022-07-19

**Authors:** Franziska Burger, Mark A. Neerincx, Willem-Paul Brinkman

**Affiliations:** ^1^Department of Intelligent Systems, Delft University of Technology, Delft, Netherlands; ^2^Department of Perceptual and Cognitive Systems, Netherlands Organisation of Applied Scientific Research (TNO), Soesterberg, Netherlands

**Keywords:** conversational agent, thought record, automated feedback, natural language processing, cognitive therapy, feasibility

## Abstract

E-mental health for depression is increasingly used in clinical practice, but patient adherence suffers as therapist involvement decreases. One reason may be the low responsiveness of existing programs: especially autonomous systems are lacking in their input interpretation and feedback-giving capabilities. Here, we explore (a) to what extent a more socially intelligent and, therefore, technologically advanced solution, namely a conversational agent, is a feasible means of collecting thought record data in dialog, (b) what people write about in their thought records, (c) whether providing content-based feedback increases motivation for thought recording, a core technique of cognitive therapy that helps patients gain an understanding of how their thoughts cause their feelings. Using the crowd-sourcing platform Prolific, 308 participants with subclinical depression symptoms were recruited and split into three conditions of varying feedback richness using the minimization method of randomization. They completed two thought recording sessions with the conversational agent: one practice session with scenarios and one open session using situations from their own lives. All participants were able to complete thought records with the agent such that the thoughts could be interpreted by the machine learning algorithm, rendering the completion of thought records with the agent feasible. Participants chose interpersonal situations nearly three times as often as achievement-related situations in the open chat session. The three most common underlying schemas were the Attachment, Competence, and Global Self-evaluation schemas. No support was found for a motivational effect of providing richer feedback. In addition to our findings, we publish the dataset of thought records for interested researchers and developers.

## 1. Introduction

Software systems increasingly help to prevent and treat depressive disorders. However, Richards and Richardson (1) have shown that the more users are left to their own devices, the higher the dropout rates. Similarly, participants in face-to-face therapy often struggle with adhering to homework assignments (2–6). We therefore explore (a) whether collecting thought record data when mimicking the conversational style of in-person care with a conversational agent is feasible, (b) what users write about in their thought records, and (c) whether offering feedback that demonstrates an understanding of the situation in response to textual input is motivating.

Depression poses a serious liability to global public health: it has a high lifetime prevalence and takes a greater toll on people's quality-adjusted life-expectancy than many other chronic conditions (7). Although depression can be treated effectively with medication, psychotherapy, or a combination (8) and possibly even prevented entirely with a toolkit of psychotherapeutic techniques, numerous barriers to seeking and obtaining help exist (9). One way to address the resulting treatment gap (10) is with *e-mental health for depression*, delivering treatment or prevention programs *via* electronic devices. As a result of the COVID-19 pandemic, e-mental health for depression is increasingly finding its way into standard clinical practice (11).

The landscape of technology-delivered depression treatment and prevention systems is varied, ranging from video-conferencing with a counselor^1^ to fully automated software programs. A literature review on the state of the art of software systems, however, revealed that the majority of systems are low-tech implementations: most of their functional components could receive information from users but were not interpreting or reacting to this input autonomously (12). This contrasts with face-to-face counseling, in which relational *micro-skills* of the counselor are thought to lead to a better alliance (13) or better rapport (14). One such micro-skill is called *reflective listening* in the context of motivational interviewing. The counselor demonstrates understanding and empathy by paraphrasing or reflecting on what was said. The advances in various areas of information processing in recent years offer an opportunity to enrich autonomous systems with these micro-skills and observe their effects. Here, we study whether feedback that provides an interpretation of a user's textual input can suffice to motivate users.

One promising technology for use in healthcare contexts are conversational agents. Provoost et al. (15), for example, found that an agent that was simply mirroring users' mood in an ecological momentary assessment task already had an adherence-stabilizing effect. Similarly, users who received personalized messages from a conversational agent felt more *heard* by the agent and were more motivated to continue when symptoms worsened than those who did not (16). And users of Woebot (17), a chatbot for depression treatment, most frequently reported a lack of understanding by the bot as the greatest nuisance when interacting with it.

A therapeutic exercise that might benefit from support by a conversational agent is *thought recording*. It is an integral part of Cognitive Therapy (CT), an evidence-based therapy form often used for the treatment (18) and sometimes used for the prevention [e.g., the Penn Resiliency Program (19)] of depressive disorders. CT rests on the idea that understanding, challenging, and changing problematic appraisals (cognitive restructuring) will improve affect. It lends itself to the dialog format because thoughts are often thought and expressed in natural language. Thought record forms provide patients with a structured format for monitoring their feelings, thoughts, and behavior in emotionally difficult situations to gain insight into *core beliefs* or *schemas*, the underlying causative patterns of thinking. Therapists ask patients to complete thought records as close in time to the negatively experienced situation as possible and thus outside of the face-to-face sessions. Patients then bring the records to the sessions to discuss with the therapist. As a consequence, the success of CT depends on patients' homework compliance (20, 21). However, adherence to homework assignments is difficult for many patients (2–6). Since depression commonly dampens motivation and a positive outlook on the future, those with symptoms may be particularly difficult to motivate (3).

In short, conversational agents are a promising technology for supporting individuals with depression symptoms in regularly completing thought records. In this work, we explore the feasibility of providing such automated conversational support for thought recording and report on the content of the thought records. In addition, we study whether the agent giving *richer* feedback, that is, feedback demonstrating a greater understanding of the user input, has a motivational effect and whether this effect is partially explained by the *insight* gained from receiving richer feedback. Finally, Grant et al. (22) found that people with a high need for self-reflection often keep diaries, indicating that this character trait motivates them to engage in self-reflection. Those who kept diaries, though, did not necessarily have more self-insight than those who did not, showing that self-reflection does not always lead to insight. If a conversational agent aids in the step from self-reflection to insight, however, those with a high need for self-reflection might be more motivated. Based on these considerations, we hypothesize (1) that as feedback richness expands, users are more motivated to engage with the conversational agent, (2) that this link is mediated by the insight that users gain from the exercise and the feedback, and (3) that the link between feedback richness and motivation is additionally moderated by users' need for self-reflection.

## 2. Materials and methods

We developed a conversational agent for the thought recording task and let participants interact with this agent to collect thought record data and to examine the motivating effect of richer feedback. For the latter objective, we chose a double-blind, between-subjects design. The independent variable, *feedback richness*, was designed to have three levels: acknowledging the reception of user input (low), feedback of low richness plus process-related feedback concerning the amount of input provided (medium), and feedback of medium richness plus content-related feedback, i.e., giving an interpretation of the input with regard to possible underlying schemas (high). As dependent variables, we used the *number of voluntarily completed thought records* in the second session with the conversational agent as well as the *engagement in self-reflection*. In addition, the mediating variable *insight* and the moderating variable *need for self-reflection* were assessed. We obtained ethical approval from the Human Research Ethics Committee of Delft University of Technology (Letter of Approval number: 1600) and pre-registered the study on the Open Science Framework (https://osf.io/5vucg).

### 2.1. Materials

The materials, including the informed consent, data management plan, pre- and post-questionnaires, the task instructions, the scenarios, the measures, the power analysis simulation script, as well as all data relevant for the analyses and the dataset of thought records can be found in the data repository accompanying this article (23).

#### 2.1.1. Conversational agent and schema-identifying algorithm

**Figure 1:**
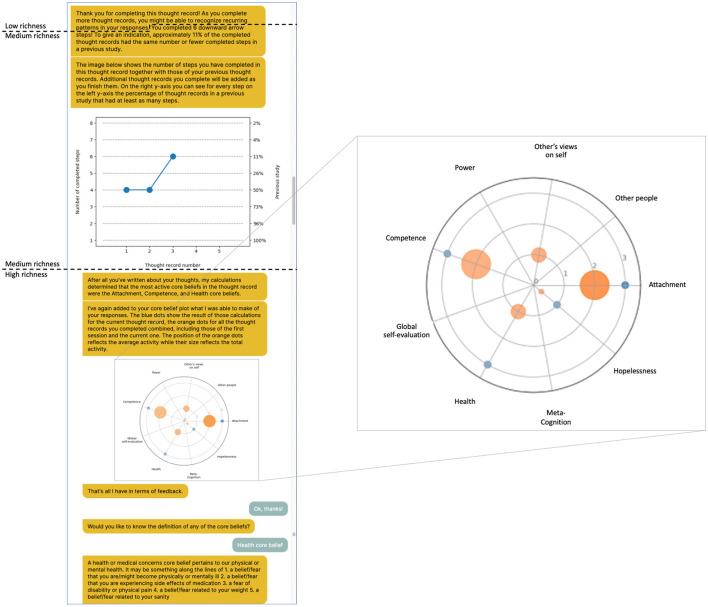
Example feedback for the third thought record in the high feedback condition. Participants in this condition saw all three feedback types combined. All participants received the first two sentences (low feedback richness). Participants in the medium feedback condition saw everything up to the end of the first plot, while participants in the high feedback condition saw also what is shown in the second plot and could optionally see the definitions for the schemas. In this thought record, three schemas were equally active and more so than the other ones as determined by the algorithm. They are shown as the blue dots on the spider plot. The activation pattern of schemas across all thought records of this participant is reflected in the size and location of the orange dots in the spider plot. New information was added to the plots after every completed thought record for the feedback of medium and high richness.

We developed the conversational agent that engaged participants in the thought record exercise using the chatbot development platform Rasa (version 2.6). The agent received a gender-neutral name (Luca). Luca had a deterministic conversational style that relied on buttons to obtain answers from the user for all interactions except within the thought record and the downward-arrow technique. The thought record form fields encompassed the four core elements of any thought record: what happened that caused the participant distress (situation), how they felt (emotion), what they thought (automatic thought), and what they did in response (behavior). In therapy, when the patient has learned to record their thoughts in this simple format, the form can be extended in various ways (24), for example, with the downward arrow technique. The agent implemented this technique by taking the automatic thought as a starting point and repeatedly asking the same question about the previously stated thought to ultimately arrive at a schema (25). In line with the technique of reflective listening, the agent gave feedback of varying levels of richness on the delineated thoughts (Figure 1). *Low feedback richness* entailed that it thanked the participants for completing the thought record and reminded them that completing more thought records might provide insight into thought patterns. *Medium feedback richness* consisted of the low-level feedback but additionally presented participants with a diagram of the number of downward arrow steps they had completed in this thought record and all previous thought records and put this number in relation to the number of people who had completed as many steps in a previous study. For *high feedback richness*, finally, the medium-level feedback was extended with natural language processing to determine one or multiple schemas that may have been activated. A spider diagram illustrated the degree to which the algorithm deemed the schema(s) present in the thought record using blue dots along nine schema axes. Orange dots in the same diagram depicted the aggregated results from previous thought records of this participant. The schemas for this condition were determined using a set of nine neural networks, one for each possible schema [see Burger et al. (26) for details concerning how the networks were trained and tested and Goodfellow et al. (27) for details concerning the statistical foundations of recurrent neural networks]. Millings and Carnelley (28) first identified and described the schemas, which were obtained from a content analysis of thought records collected from a clinical population with depression and/or anxiety. The feedback of medium richness served as a control condition for the feedback of high richness, as it allowed separating the effect of giving feedback on participants' efforts from that of giving feedback that might generate insight.

#### 2.1.2. Scenarios

The agent used a set of ten scenarios to select from for the scenario-based thought records of the first session. These were taken mostly from the Ways of Responding scale (29) with two added from the Cognitive Error Questionnaire (30). We divided the scenarios into two sets of five scenarios, one with situations that might be difficult on an interpersonal level (e.g., an acquaintance does not wave back at you) and one with situations that might be difficult on an achievement-related level (e.g., you were fired from your new job for not meeting your quota). The agent presented participants with one randomly chosen scenario from each of the two sets.

### 2.2. Measures

We used the three subscales of the Self-Reflection and Insight Scale (22) as measures: the *Engagement in Self-Reflection* subscale for self-reported motivation (outcome variable), the *insight* subscale for self-insight participants gain from thought recording with the agent (mediator variable), and the *Need for Self-Reflection* subscale for participants' general need to reflect on their thoughts, emotions, and behaviors (moderator variable). We modified the *Insight* and the *Engagement in Self-Reflection* subscales to measure state rather than trait variables. For example, the item “I am usually aware of my thoughts” (Insight) became “Completing the thought-recording task with the chatbot has made me more aware of my thoughts.”

### 2.3. Participants

Participants were recruited from Prolific, a crowd-sourcing platform for research studies. We pre-screened participants on their depression symptoms using the 9-item patient health questionnaire (PHQ-9) (31). In line with (32), we used the range of 4 < *score* < 8 for selecting *subclinical* participants unlikely to meet diagnostic criteria for depression. A clinical population was not chosen for ethical reasons and a healthy population was not chosen because we expected a subclinical population to be more similar to a clinical population in terms of motivational barriers. Participants were not informed of their score or the selection criterion. To participate in the pre-screening, participants had to be at least 18 years of age and fluent speakers of English. We recruited 2899 participants. Participants with subclinical depression symptoms and those who did not fail more than one attention check (519 participants) were invited to participate in the next part of the study. With a power analysis simulation following the bias-corrected bootstrapping method (33) modified for a categorical predictor and a Poisson-distributed outcome variable, we determined that 306 participants would be needed for a medium effect size [in line with (34), at least 13% of the variance in *feedback richness* estimated to be explained by *insight*, a-path, and at least 13% of the variance in *motivation* estimated to be explained by *insight* when controlling for *feedback richness*, b-path] at α =.05 and power of 80%. We stopped recruitment after having complete data of 306 participants, but, due to participants still being in the pipeline when recruitment stopped, the final dataset contains the data of 308 participants (143 female, 164 male, 1 other). Their ages ranged from 18 to 75 with *mean*_*age*_ = 30.97 and *SD*_*age*_ = 11.66. Participants could be excluded for failing multiple attention checks, failing multiple instruction comprehension questions, not taking the task seriously (writing gibberish, copying and pasting content from other websites, writing incoherent responses to the agent), or technical problems. In total, 36 participants had to be excluded for one of these reasons of which only one was excluded for not taking the task seriously.

### 2.4. Procedure

In the first part of the experiment, Prolific redirected participants to the survey tool Qualtrics to complete the *Need for Self-Reflection* scale (pre-questionnaire). Based on the result, Qualtrics divided them into one of three possible buckets (low, medium, and high need for self-reflection). Within a few hours after completing the pre-questionnaire, a message on Prolific invited participants to the next part of the experiment, which consisted of instructions and the first thought recording session with the conversational agent. Participants were blindly assigned to the experimental conditions using the minimization method of randomization (35) with the need for self-reflection buckets as the only variate. The agent started the conversation in the first session with a brief onboarding message. It then repeated the main instructions. Upon presenting the first scenario to the user, it proceeded with the thought record and downward arrow form fields. Finally, it gave feedback depending on the condition and asked if the participant was ready for the next scenario. The first session always consisted of two scenario-based thought records to become familiar with the task and the feedback. Participants received an invitation via Prolific to participate in the second session between 24 and 48 hours after completing the first session. The second session proceeded as the first but with the agent moving directly to the thought record after an initial “welcome back" exchange. In the second session, the agent asked participants to complete at least one but as many additional thought records as they wanted. For the thought records of this session, they were taking day-to-day situations from their own lives. We compensated each session with 2 GBP based on an estimated completion time of 20 min. No extra monetary compensation was provided for more completed thought records to not interfere with motivation. Participants were informed at the beginning of the second chat session of the expected completion time for the post-questionnaire, which included the *Insight* and *Engagement in Self-Reflection* scales as well as any additional comments or feedback. In total, participants could receive 4.3 GBP for completing all parts of the study.

### 2.5. Data and analysis method

To determine feasibility, we correlated the nine values of the frequency distribution over the schemas as assigned by the algorithm on this dataset with the distribution of two previously collected datasets (26, 28). To this end, we recoded the labels for each utterance from ordinal (0–schema not present to 3–schema clearly present) to binomial (0–schema not present and 1–schema at least a little bit present). The same procedure was followed for the dataset of Burger et al. (26). For the Millings and Carnelley (28) dataset, however, we compare with the frequencies reported in the article, which are based on entire thought records rather than utterances and which were manually assigned.

Two independent coders (one male and one female computer science student) labeled all thought records of the second session using the DIAMONDS framework for psychologically relevant situation characteristics (36) to examine the content of participants' thought records in the second session. They were trained on ten example thought records in a joint session of 1 hour to clarify the definitions of the DIAMONDS. Since participants were asked to report only situations that caused a negative emotion, we dropped the Positivity characteristic. Two further labeling categories were added: *COVID19-related* and *situation type (achievement-related vs. interpersonal)*. All labels, were binomial (situation *has* or *does not have* characteristic). Coders were instructed together and coded 10 example situations (taken from the first chat session) together with the first author before coding the situations described by participants in the second chat session independently. While interrater agreement was mixed on the DIAMONDS, ranging from minimal κ = 0.25 (Negativity) *via* moderate κ = 0.66 (Interpersonal) to strong κ = 0.83 (Mating), the raters largely agreed on the frequency of labels within the dataset (Pearson *r* = 0.89 based on 10 values).

The reliability of the three subscales of the Self-Reflection and Insight scale was good (*Need for Self-Reflection*: Crohnbach's α = 0.85 with [0.85, 0.86] 95% CI) and acceptable (*Insight*: α = 0.76 with [0.75, 0.77] 95% CI and *Engagement in Self-Reflection*: α = 0.75 with [0.73, 0.76] 95% CI). The items of each subscale were summed to obtain a summary score for the variables *need for self-reflection, insight*, and *engagement in self-reflection*. Engagement in self-reflection was negatively skewed (ceiling effect) and we consequently boxcox-tranformed the data with λ = 1.97 for use in the analyses.

We followed the Baron and Kenny method (37) to test for the mediated effect. For the direct effect, this entailed fitting a generalized linear model with a log-link function as the behavioral outcome variable *number of voluntarily completed thought records* was expected to be Poisson-distributed and fitting a second linear model for the self-report outcome variable *engagement in self-reflection*. A further linear model was fit to test whether *feedback richness* affected *insight* (mediator). Finally, we fit one generalized linear model and one linear model to assess the effect of the mediator on each of the two outcome variables. Due to the lack of mediation observed in these models, we did not test for moderated mediation. However, we checked with two linear regression models whether participants' *need for self-reflection* (moderator) moderated the direct link between the *feedback richness* and either of two outcome variables.

## 3. Results

All 308 participants were able to complete thought records with the conversational agent. Of the 93 participants who chose to comment, 34 reported that they found the experiment insightful, with five participants specifically mentioning the added value of the agent and the feedback (“The chatbot makes the experience more friendly,” “shows that chatbot can offer a sincere alternative to human response,” “[...] get immediate feedback than on a paper which feels sometimes too much like homework,” “[...] I felt like someone was paying attention to me,” “this chatbot is really helpful in discovering my thought patterns”). However, another five participants also commented that they struggled with the downward arrow technique and would have liked more agent or even human support (“I know it's a chatbot, but I wish Luca could engage a little more when trying to work your way down the arrow,” “it was really hard to go down the thought steps instead of in circles, i feel like maybe a human would've been able to help with that,” “I also would prefer to do this activity with a real person rather than a chatbot,” “It is not always easy to figure out what the next drill down should be,” “Was somewhat confused to break my thought patterns down in the arrow scheme though”). Only two participants remarked negatively about the rich feedback (“I think Luca's overall assessment of my core beliefs was decent, but not perfect” and “I found the circular diagram a bit difficult to understand”) and one in the low-level feedback condition about the lack thereof (“I did not see any feedback from the chatbot, it would be nice to”).

**Figure 2:**
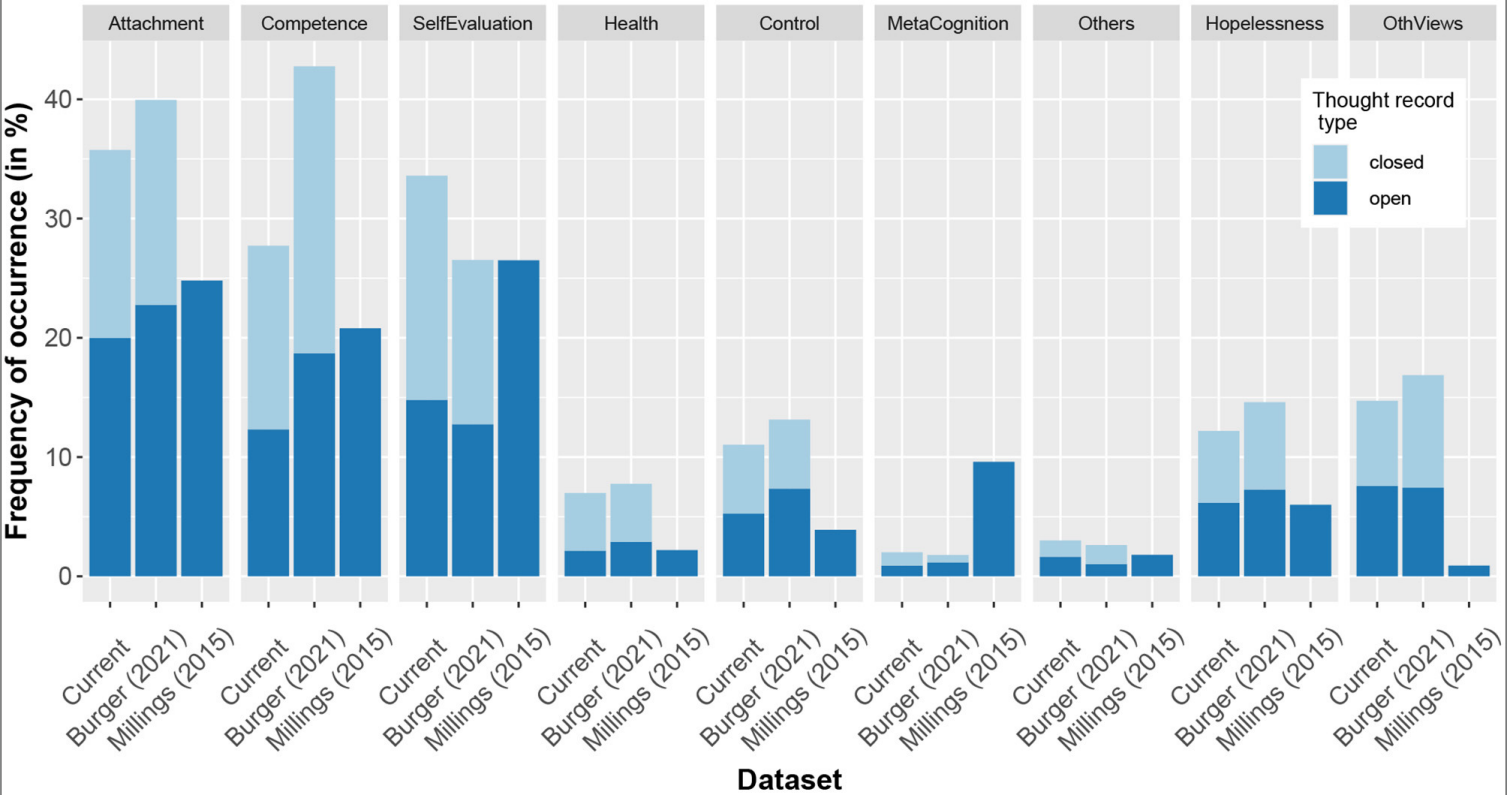
Frequency of occurrence of schemas in this dataset compared to a previously collected dataset (26), in which participants completed thought records in survey format, and that of Millings and Carnelley (28). In the current dataset and the one collected by Burger et al. (26) schemas are identified by an algorithm from thoughts (automatic thought or any downward arrow step), while in the dataset by Millings and Carnelley (28), schemas were identified by the authors and from entire thought records. While the algorithm assigns ordinal codes corresponding to the degree to which a schema was present, for the purpose of this analysis, we recoded these scores to binomial scores with all values above 0 being coded as 1. *Closed* thought records are those based on scripted scenarios while *open* thought records are those in which participants report on situations from their lives.

The relative frequency distribution with which the schema-labeling algorithm identified certain schemas in this dataset compared to that of the previous study by Burger et al. (26) correlated highly for both the scenario-based (Spearman's ρ = 0.93) and the personal thought records (Spearman's ρ = 0.95). The schema frequency distribution of both scenario-based and personal thought records taken together also correlated positively (Spearman's ρ = 0.57) with that reported by Millings and Carnelley (28) (Figure 2). Across all three datasets, the most frequently occurring schemas were the Attachment, Competence, and Global Self-Evaluation schemas.

We present a typical thought record situation for each content label in Table 1. Besides reporting mostly negative situations (98% Negativity), participants opted for more interpersonal and social than achievement-related or intellectual situations.

**Table 1:** Example thought record situations for each content label.

**Label**	**Explanation**	**IRR (**κ**)**	**MRF (%)**	**Example situation**
Achievement-related	Situations in which self-esteem is at risk because it is possible to perform poorly.	0.58	19	When I didn't get a job I was interviewed for.
Interpersonal	Social situations that can affect one's self-worth.	0.66	60	My colleague blamed me for their mistake.
COVID-related	Thought records in which participants mention COVID-19.	0.83	4	Staying indoors a lot due to the pandemic.
Duty	Situations that require executing a task conscientiously or dutifully.	0.43	27	I had to give a presentation.
Intellect	Situations that are cognitively stimulating.	0.46	19	I was worried about sitting an exam for university.
Adversity	Situations in which one is criticized, blamed, or dominated.	0.58	21	I was really sick and my then-boss made me work while I was sick.
Mating	Situations that involve potential or actual romantic partners.	0.83	19	My husband is stressed and moody because of it.
Negativity	Situations that are anxiety-inducing, stressful, frustrating, upsetting.	0.25	98	I rejected a holiday job offer because it paid too little and now I cannot find anything else.
Deception	Situations that can result in feelings of hostility due to deception or sabotage.	0.35	13	I found out I was being cheated on by my girlfriend.
Sociality	Situations that involve social interaction.	0.32	48	I was given a huge amount of rudeness and grief by a customer at work.

*The column IRR shows the InterRater Reliability while the column MRF shows the Mean Rater Frequency, i.e. the mean of how frequently the raters found a specific label to occur in the dataset. Labels were not mutually exclusive*.

**Figure 3:**
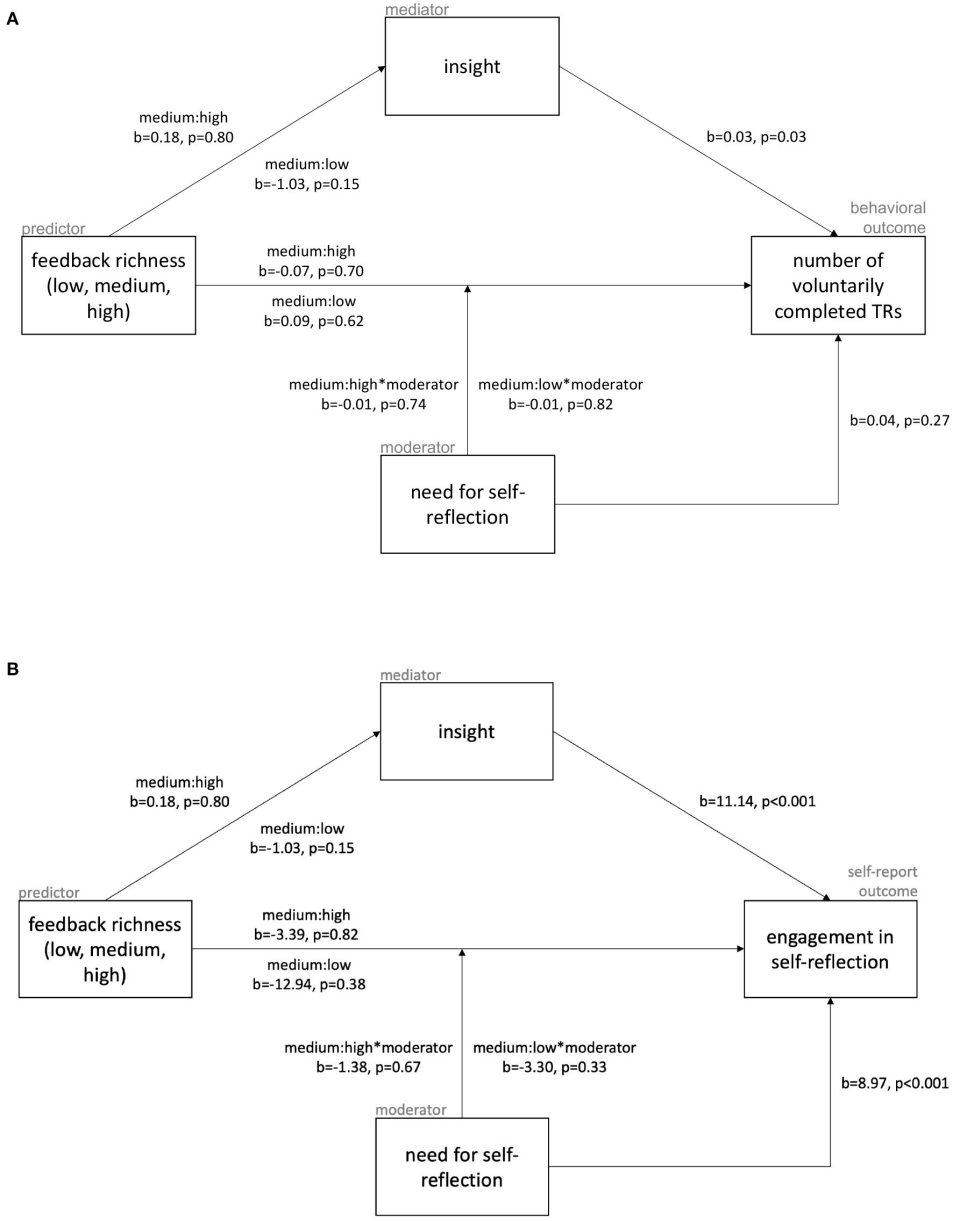
Results of all paths of the mediation and moderation analyses, with the behavioral outcome measure of motivation, number of voluntarily completed thought records in the second chat session, shown in **(A)** and the self-reported one, boxcox-tranformed (λ = 1.97) engagement in self-reflection, shown in **(B)**.

Participants felt engaged in self-reflection when completing the thought records (*mean* = 29.56, *SD* = 4.04), but completed, on average, only 1.62 (*SD* = 0.72) thought records in the second session. The direct effect of *feedback richness* on either of these measures of motivation (Figure 3) was not observed. There was also no effect found for the *feedback richness* on the mediator variable *insight* (a-path). As a consequence, partial mediation was no longer relevant. Nonetheless, a significant link between the mediating variable *insight* was found for both measures of motivation (b-path): for every additional scale point of insight they report, participants complete 1.03 times as many thought records [*b* = 0.03, *z*_(304)_ = 2.12, *p* = 0.03] and feel 4.91 scale points more engaged [*b* = 11.14, *t*_(304)_ = 11.30, *p* < 0.001] on a scale ranging from 6 to 36.

The moderator *need for self-reflection* had no effect on the direct link between *feedback richness* and either of the two motivation measures. Additionally, participants' need for self-reflection did not predict how many thought records they would do voluntarily. It did, however, explain their engagement in the task with participants feeling 4.42 scale points more engaged with every additional scale point of their self-reported need for self-reflection [*b* = 8.97, *t*_(302)_ = 3.69, *p* < 0.001].

## 4. Discussion

The findings show that thought recording with a conversational agent is feasible for a subclinically depressed population: 100% of participants completed the thought records such that the machine learning algorithm trained on a similar dataset could label thoughts with regard to the underlying schemas. The distribution of the thus assigned schema labels not only closely resembles that of the dataset used for training the algorithm (healthy population) but also the manually labeled dataset of Millings and Carnelley (28) (clinical population). In addition, participants frequently reported enjoying the experiment and finding it so valuable that they intended to continue using the technique in their day-to-day lives. Prior studies looking into the feasibility of using conversational agents for mental health interventions have found similar results concerning user satisfaction and ability to interact with the conversational agents (38, 39), but none had specifically studied thought record completion before. In terms of content, participants' personal thought records concerned interpersonal (58%) or social situations approximately three times more often than achievement-related (19%) or intellect-related ones, which is also reflected in the schemas, with the *Attachment* schema being identified by the algorithm more than the *Competence* schema. Despite around 4% of situations mentioning the COVID-19 pandemic, the *Health* schema was not more active in this dataset than in the ones collected before the pandemic. This is likely due to the training dataset of the algorithm being biased toward dieting situations for this particular schema. It can also be seen from the frequency of the *Negativity* label that participants were able to choose negative situations (98%) as instructed but sometimes put a positive spin on the meaning of the situation for themselves (e.g., “It says that I don't have to feel obliged to do anything for anyone, and I don't want to feel that way”). It is important to note here, however, that the participants were subclinically depressed and these findings concerning the feasibility and content of the thought records may not generalize to a clinical population.

We did not observe the hypothesized effect of feedback on motivation: the results did not show that the *feedback richness* influenced either the *motivation* of participants (direct effect) or the hypothesized mediating variable *insight participants gained from the task*. However, participants' gained insight positively related to both measures of motivation, and participants who reported a greater need for self-reflection also reported being more engaged in the task. When regarding these findings, limitations of the feedback on the one hand and of motivation on the other should be considered. For one, the spider plot and the academic definitions of the schemas in the rich-feedback condition might not have been as accessible as we had hoped and therefore did not add the expected value. This could be addressed in future research by following an cyclic design approach including both end users and graphical designers, simplifying the feedback, including measures of graphic literacy and health literacy as moderators, or conducting pilot studies to determine whether feedback is processed as desired. In line with this, articles concerning the design of graphical feedback in behavior change support systems argue for the importance of health literacy and usability as guiding principles (40, 41), and platforms that have successfully used complex informational feedback in graphical format have done so in collaboration with a design company and with an iterative refinement process (42). Another possible limitation of the feedback is that participants may have perceived the richer feedback as discrepant with the otherwise limited conversational capabilities of the agent. As far as motivation is concerned, this was measured with just one session, such that small issues like participants misclicking, minor technical glitches, or external disturbances may have played a larger role than in a long-term study. Additionally, motivation may also have been adversely affected by the monetary compensation in the online context and may have panned out differently with patients being internally motivated by a desire to get healthy. Lastly, our participant sample included more males than females, which is noteworthy due to depression being more prevalent in women. Future research might therefore consider looking into a moderating effect of gender. When looking more closely at the distribution of schemas in the different populations (Figure 2), the clinical sample differs most markedly with respect to the *Global self-evaluation*, the *Meta-Cognition*, and the *Other's views on self* schemas. Since self-evaluation and meta-cognition are likely to also be linked to one's need for self-reflection and one's engagement in self-reflection, it is possible that the results would play out differently in a clinical sample. Since the experiment was not underpowered, however, and some limitations pertain to all three conditions, we conclude from the null results that this type of feedback richness is unlikely to have a large effect on motivation regardless of the limitations.

In summary, people with subclinical depression symptoms are capable of thought recording with a conversational agent. Not only were the thoughts they recorded of sufficient richness to allow for automatic schema identification, but the three most frequently occurring schemas (Attachment, Competence, and Global Self-evaluation) in this sample of subclinically depressed participants were the same as in previous work with healthy (26) and with clinical (28) populations. However, no support could be found that richer feedback leads to a higher motivation to engage with thought recording. More research and perhaps participatory design are needed to determine engagement strategies for the agent that can lead to greater adherence. One possible route to explore is to combine the content-based feedback generated by reflective listening with additional communication strategies of motivational interviewing, such as establishing rapport or eliciting self-motivational messages (43). Finally, the study could be repeated with a clinical population to determine the role that other (de-)motivational forces, such as dampened enjoyment of tasks and the wish to get healthy, play in this population. We contribute the dataset of collected thought records and all measures for researchers and developers interested in working with this data.

## Data availability statement

The original, anonymized data that were collected in this research study are available in a publicly accessible 4TU.ResearchData repository. All materials and analyses to reproduce the results or the study can be found in the same repository. The repository is registered under doi: 10.4121/20137736.

## Ethics statement

The studies involving human participants were reviewed and approved by Human Research Ethics Committee Delft University of Technology. The participants provided their written informed consent to participate in this study.

## Author contributions

FB and W-PB closely collaborated on the drafting and planning of the conversational agent, the experiment, the analysis of the data, and the article. FB developed the conversational agent, conducted the experiment, analyzed the data, and created all figures. W-PB and MN supervised the entire research process, providing critical feedback and guidance throughout. FB, W-PB, and MN jointly wrote the article. All authors contributed to the article and approved the submitted version.

## Funding

This work was funded by two projects of the Dutch 4TU center for Humans & Technology: the Smart Social Systems and Spaces for Living Well (S4) project and the Pride and Prejudice project.

## Conflict of interest

The authors declare that the research was conducted in the absence of any commercial or financial relationships that could be construed as a potential conflict of interest.

## Publisher's note

All claims expressed in this article are solely those of the authors and do not necessarily represent those of their affiliated organizations, or those of the publisher, the editors and the reviewers. Any product that may be evaluated in this article, or claim that may be made by its manufacturer, is not guaranteed or endorsed by the publisher.

## Footnotes

^1^We use the term *counselor* here to subsume primary care providers, coaches, counselors, and therapists.
